# Multiple conserved states characterize the twist landscape of the bacterial actin homolog MreB

**DOI:** 10.1016/j.csbj.2022.10.008

**Published:** 2022-10-07

**Authors:** Benjamin D. Knapp, Michael D. Ward, Gregory R. Bowman, Handuo Shi, Kerwyn Casey Huang

**Affiliations:** a Biophysics Program, Stanford University, Stanford, CA 94305, USA; b Department of Biochemistry & Molecular Biophysics, Washington University School of Medicine, St. Louis, MO 63130, USA; c Center for the Science and Engineering of Living Systems, Washington University in St. Louis, St. Louis, MO 63130, USA; d Department of Microbiology and Immunology, Stanford University School of Medicine, Stanford, CA 94305, USA; e Department of Bioengineering, Stanford University, Stanford, CA 94305, USA; f Chan Zuckerberg Biohub, San Francisco, CA 94158, USA

**Keywords:** Chiral twist, Bacterial cytoskeleton, Actin homologs, Molecular dynamics, Deep learning, DiffNets

## Abstract

Filament formation by cytoskeletal proteins is critical to their involvement in myriad cellular processes. The bacterial actin homolog MreB, which is essential for cell-shape determination in many rod-shaped bacteria, has served as a model system for studying the mechanics of cytoskeletal filaments. Previous molecular dynamics (MD) simulations revealed that the twist of MreB double protofilaments is dependent on the bound nucleotide, as well as binding to the membrane or the accessory protein RodZ, and MreB mutations that modulate twist also affect MreB spatial organization and cell shape. Here, we show that MreB double protofilaments can adopt multiple twist states during microsecond-scale MD simulations. A deep learning algorithm trained only on high- and low-twist states robustly identified all twist conformations across most perturbations of ATP-bound MreB, suggesting the existence of a conserved set of states whose occupancy is affected by each perturbation to MreB. Simulations replacing ATP with ADP indicated that twist states were generally stable after hydrolysis. These findings suggest a rich twist landscape that could provide the capacity to tune MreB activity and therefore its effects on cell shape.

## Graphical abstract

**Figure:**
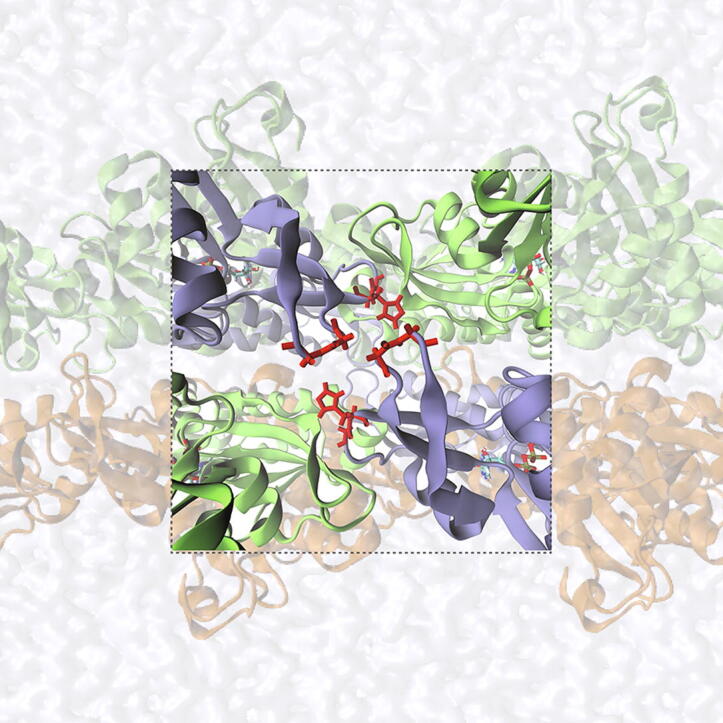

## 1 Introduction

Many bacteria encode homologs of the major eukaryotic cytoskeletal proteins actin and tubulin, with diverse biological functions including morphogenesis [51], [54], division [1], [29], [45], DNA segregation [16], and more [6], [50]. In both eukaryotes and prokaryotes, the function of many cytoskeletal proteins relies on their ability to form filaments, in order to exert forces [32], transport material [22], position organelles [26], and detect geometry [13]. While crystallography has proven a powerful tool for revealing the vast array of filament structures adopted by bacterial cytoskeletal proteins [29], [45], [51], [52], it is challenging to infer function from static structures. Molecular dynamics (MD) simulations provide insights into the potential for conformational changes at multiple scales, enabling determination of important degrees of freedom [8], changes to filament interfaces [19], and quantification of the mechanical properties of filaments [10], [19].

In many rod-shaped bacteria such as *Escherichia coli*, MreB is an essential protein that dictates cell shape via regulation of new cell wall synthesis by localizing in a curvature-dependent manner [41], [48]. MreB forms short, dynamic filaments that move along the cell periphery [54]. While MreB movement is approximately circumferential, the directionality is chiral (has a preferred handedness relative to the longitudinal axis of a cell), and the degree of chirality is related to cell-body twist during elongation [12], [46], [55]. RodZ directly binds to [3] and moves with MreB [34], and RodZ regulates cell shape by modulating MreB curvature sensing [9], [21], [35]. Mutations in MreB can lead to dramatic changes in cell morphology and/or cell size, potentially through altered curvature localization patterns [18], [25], [33], [42], suggesting that MreB is a morphological tuning knob with a wide dynamic range. However, the structural properties that dictate how perturbations to MreB alter its curvature-sensing preferences and generate shape changes has remained mysterious.

Like eukaryotic actin, MreB has a U-shaped four-domain structure, with two beta domains and a nucleotide binding pocket between two alpha domains [24]. Previous MD simulations of *Thermatoga maritima* MreB revealed nucleotide- and polymerization-dependent conformational changes of MreB monomers [8] that were later observed using X-ray crystallography [52]. Simulations also predicted that intra-domain opening is connected to bending of a single protofilament [8]. Crystal structures revealed that *Caulobacter crescentus* MreB (CcMreB) forms anti-parallel double protofilaments [52], and MD simulations exploring the dynamics of double protofilaments over ∼100 ns demonstrated the potential for left-handed twist, whose degree depends on the bound nucleotide as well as binding to RodZ and/or the membrane [43]. Simulations also predicted that point mutations in MreB can change the intrinsic twist, and changes in twist angles correlated with MreB filament conformation *in vivo* [43], suggesting that twist is a key variable linking the activity of MreB to cell shape. While simulation results were largely reproducible, some systems also showed occasional, large fluctuations [43], indicating that longer simulations are necessary to shed light on the stability of MreB twist states.

Anton2 is a special purpose supercomputer for molecular dynamics calculations, enabling simulations at microsecond time scales [40]. These longer simulations provide the potential for proteins to traverse multiple conformational states [14], [15], thereby providing a more comprehensive and physiologically relevant picture connecting protein structure and dynamics to function. Motivated by the connections between MreB twist and cell shape, we sought to examine whether the seemingly equilibrated twist states in previous simulations were a complete representation of the twist landscape by performing microsecond-scale simulations of CcMreB double protofilaments. We found that MreB can adopt multiple twist states that can remain stable over hundreds of nanoseconds, and perturbations to MreB alter the relative occupancy of these states while conserving the distinct twist states. A deep learning method revealed structural determinants of state differences and identified residues located at the filament interface that are key to twist state classification. These findings showcase the potential of microsecond-scale MD simulations to quantify the structural and mechanical landscapes of cytoskeletal proteins.

## 2 Results

### 2.1 Double protofilaments fluctuate among multiple states with quasi-stable twist angles

**Fig. 1:**
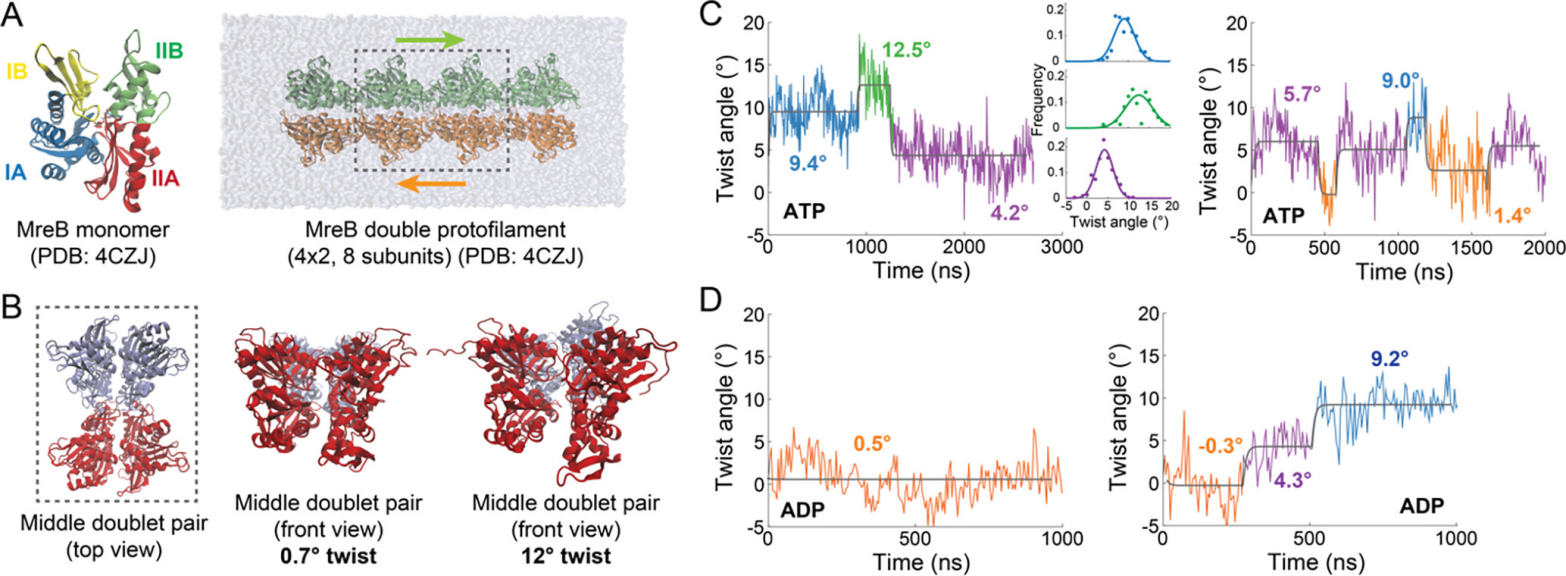
MreB double protofilaments adopt quasi-stable twist states with similar sets of angles across simulations and nucleotide-binding states. A)   Left: *Caulobacter crescentus* MreB (CcMreB) monomer structure bound to ATP (from filament structure PDB ID: 4CZJ). The four subdomains align with those of actin and are defined by IA (residues 9 to 36 and residues 322 to 345, blue), IB (residues 37 to 81, yellow), IIA (residues 151 to 186 and residues 265 to 321, red), and IIB (residues 187 to 264, green). Right: double protofilament (4x2) structure of CcMreB (PDB ID: 4CZJ), which consists of antiparallel MreB strands (green, orange). The middle doublet (dotted square) was used for twist measurements (Methods). The silver transparent water box is shown to illustrate the size of the simulated system. B)   The twist angle of an MreB double protofilament measures the relative rotation of pairs within the middle doublet. Left: schematic of the middle doublet (2x2) used to calculate the twist angle of MreB double protofilaments. Shown in red and blue are antiparallel subunit pairs. Middle: subunit pairs (red, blue) are shown through the long axis of the double protofilament from the crystal structure (0.7° twist). Right: subunit pairs (red, blue) rotate in opposite directions to generate twist, shown here at 12°. C)   Twist angle varied throughout ATP-bound double protofilament simulations. Left: twist angle throughout a 2.7-ms simulation of an ATP-bound 4x2 MreB structure. Colored segments with overlapping horizontal grey lines represent states identified by the change-point algorithm Steppi (Methods). Inset: the twist angle distribution of each state fit with a Gaussian function. Right: A 2-ms replicate simulation of an ATP-bound 4x2 MreB filament displayed different twist dynamics from the simulation on the left, but the collective set of twist angles was similar in the two simulations. Steppi-identified states with similar mean values between the two simulations are plotted with the same color. D) Twist angle sometimes varied throughout ADP-bound double protofilament simulations and adopted generally lower values than ATP-bound structures. Left: A 1-ms simulation of an ADP-bound 4x2 MreB double protofilament was characterized by a single state with low twist (orange). Right: the twist angle of a replicate 1-ms simulation of an ADP-bound 4x2 MreB double protofilament varied between three states.

We previously showed that MreB double protofilaments exhibit a nonzero twist angle that is stable over ∼100 ns but dependent on the bound nucleotide [43]. To test whether twist angle is stable over longer time scales, we carried out µs-scale MD simulations on Anton2 using a 4x2 double protofilament initialized with the crystal structure (PDB ID: 4CZF; Fig. 1A). To avoid effects due to greater fluctuations of the filament ends, we quantified the twist angle based on the middle doublet pair (Fig. 1B, Fig. S1A, Methods). We first simulated an ATP-bound double protofilament for >2.5 µs (Fig. 1C, left). At the beginning of the simulation, the double protofilament quickly adopted a twist angle of ∼10° and maintained this value for almost 1 µs. These twist angles were consistent with those in our previous, shorter simulations of ATP-bound MreB [43]. However, the twist angle then quickly increased to >12° over an interval of <5 ns and remained around this value for >300 ns. After ∼1200 ns, the twist angle decreased to <5° within 200 ns and remained low for the remainder of the simulation. The ∼5° twist angle was more reminiscent of our previous, ∼100-ns simulations of ADP-bound MreB [43]. The distribution of twist angles within each of these intervals (excluding boundaries to avoid the transitions) was approximately Gaussian (Fig. 1C, insets), suggesting that the double protofilament switched between quasi-stable states. Consistent with this picture, the Steppi algorithm, which is an information-based change-point analysis algorithm for detecting transitions between discrete states [27], [57], identified three states defined by the boundaries described above (Fig. 1C, left).

Having observed such transitions in twist angle, we carried out another, independent simulation of a 4x2 ATP-bound double protofilament. In this simulation, the twist angle initially started at ∼5°, similar to the final state of the first simulation (Fig. 1C, left), and fluctuated around this value for almost 500 ns (Fig. 1C, right). After that point, like the first simulation, twist angle exhibited several rapid transitions. A Steppi analysis of the twist trajectory suggested the existence of twist states with low (∼0°), intermediate (∼5°), and high (∼10°) values. This simulation supports the hypothesis that an ATP-bound structure can access quasi-stable states with both higher twist angles and lower twist angles more similar to the expected twist of ADP-bound double protofilaments.

To determine the stability of ADP-bound MreB, we carried out two, independent 1-µs simulations. In both simulations, the twist angle initially fluctuated around 0°. In the first simulation, the twist angle remained low for the entire 1 µs; Steppi predicted a single state (Fig. 1D, left). By contrast, in the second simulation, after 300 ns the twist angle rapidly increased to ∼5°, and then rapidly increased to ∼10° after 500 ns (Fig. 1D, right). Steppi predicted three states, the first of which exhibited similar average twist to that of the first ADP-bound simulation (Fig. 1D, left) and the last two with similar twist to that of the initial and final states in the first ATP-bound simulation (Fig. 1C, left).

To determine if other filament conformational changes accompanied twist-state transitions, we evaluated bending dynamics throughout these trajectories. While bending and twist angles (Fig. S1A) were largely uncorrelated in ATP- or ADP-bound filament simulations (Fig. S1B,C), we identified a weak negative correlation between the out-of-plane bending angle and the twist angle in both ATP simulations (Fig. S1B), suggesting a slight trade-off between twist and bending angles. This relationship was evident in only one of the ADP-bound filament simulations (Fig. S1C). Steppi predicted only a single state transition of the out-of-plane bending angle in each ATP simulation, despite the identification of multiple twist-state transitions (Fig. 1C, S1D). Thus, while filament bending decreases with twist angle in ATP-bound filament simulations, twist angle appears to be more robustly connected to state transitions.

We also evaluated filament stability throughout these simulations by measuring the buried solvent-accessible surface area (bSASA, Methods) across all doublet pairs, which measures interaction surfaces inaccessible to water. We found that the bSASA was highly stable across both ATP- and ADP-bound filament simulations, with no obvious signature during intervals associated with twist transitions (Fig. S2A). In addition, twist angle was only weakly anticorrelated with the bSASA of the middle doublet (the filament section from which twist was computed) (Fig. S2B). Thus, high twist states are accessible without substantial cost to filament stability, and filaments remain stable throughout twist-state transitions.

**Fig. 2:**
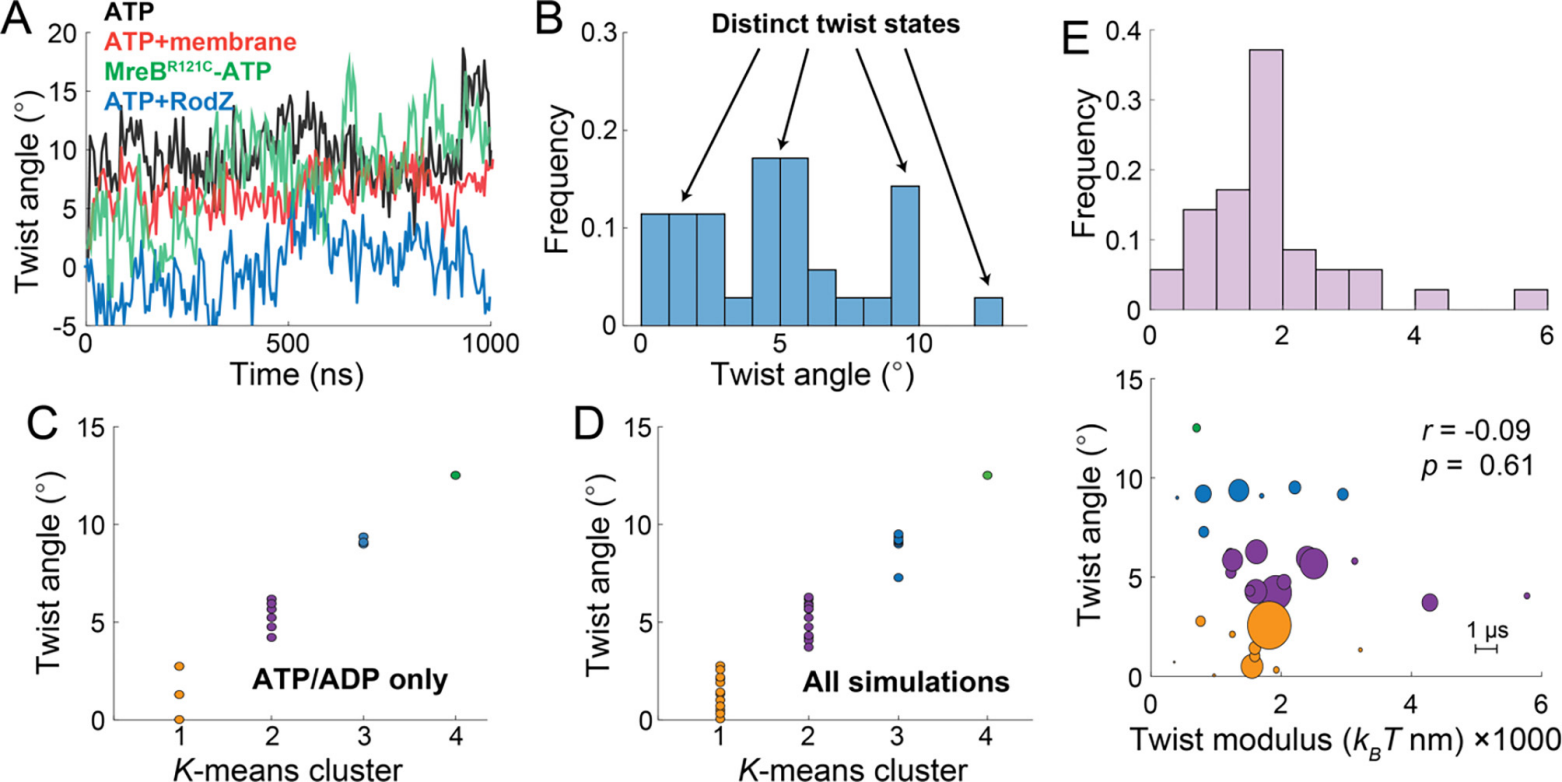
MreB double protofilaments occupy at least four distinct twist states and perturbations shift their relative occupancy. A)   Representative 1-ms trajectories of the effects of each perturbation on MreB double protofilament twist behavior. Binding to a membrane (red) decreased twist, while the R121C mutation led to higher twist angles (green). Binding to RodZ (blue) caused the largest reduction in twist angle. B)   The distribution of mean twist angles among Steppi-identified states across all simulations from this study shows multimodal behavior. Modes are identified with arrows. C)   *K*-means clustering with *n*=4 of Steppi-identified twist states across ATP- and ADP-bound free (no membrane, no RodZ) double protofilament simulations. Clusters are identified by color and each state instance is shown as a colored circle. D)   *K*-means clustering with *n*=4 of Steppi-identified twist states across all simulations. Clusters are identified by color and each state instance is shown as a colored circle. E) Top: distribution of twist moduli calculated for each state identified by Steppi (Methods). Bottom: twist modulus did not display any obvious trends across twist states. States are colored based on *k*-means cluster (C,D), and the size of each circle is proportional to the state lifetime (scale shown at bottom right).

These simulations suggest the presence of multiple twist states of MreB double protofilaments and highlight the importance of µs-scale simulations for exploring the twist landscape of MreB.

### 2.2 Perturbations to MreB shift twist angles among similar sets of states

We noticed that the quasi-stable twist states adopted by ATP-bound double protofilaments (Fig. 1C, S3A) were quantitatively similar to those of ADP-bound double protofilaments (Fig. 1D, S3B). In our previous study [43]), we predicted using ∼100 ns-scale MD simulations that membrane binding suppresses ATP-bound double protofilament twisting and that mutations in MreB and binding to RodZ modulate intrinsic twist. To determine how these perturbations affect twist on longer time scales, we carried out µs-scale simulations of double protofilaments bound to a membrane patch, bound to RodZ, and with the MreB^R121C^ mutation (in solution and bound to a membrane patch), all bound to ATP, and carried out two replicates of each lasting 1 µs or longer (Table S1, Fig. S3A-D). During the initial period of each simulation, our results were generally consistent with our previous study [43]. After binding to the membrane, the twist angle was predominantly ∼5° in both replicates, only occasionally increasing to ∼10° or decreasing to ∼0° (Fig. 2A, red; Fig. S3A). One MreB^R121C^ simulation initially exhibited low twist values (0-5°) in solution for the first 100 ns, which then increased and stabilized at larger values close to ∼10° (Fig. 2A, green; Fig. S3C); this larger twist was suppressed by membrane binding (Fig. S3C). RodZ binding reduced twist angle, with long intervals at 0° or 5° and even some periods with negative twist angle (right-handed twist in our formalism; Fig. 2A, blue; Fig. S3D). In each of these cases, Steppi predicted the existence of multiple states. To quantify the overall twist landscape of MreB, we combined all our datasets (Fig. S3) and calculated the histogram of mean twist values across all Steppi-predicted states. Consistent with our visual inspection, the twist angle distribution exhibited four peaks, corresponding to ∼0°, 5°, 10°, and 12.5° (Fig. 2B); the distribution was qualitatively similar if each state was weighted by the time of occupancy (Fig. S4A). *K*-means clustering with four clusters based on wild-type ATP- or ADP-bound double protofilaments alone resulted in groups with twist angles of ∼0°, 5°, 10°, and 12.5° (Fig. 2C, S4B; Methods), as did clustering of all simulations (Fig. 2D, S4C). These results suggest that the perturbations to MreB are not altering the twist states themselves, but rather shifting their relative occupancy.

**Fig. 3:**
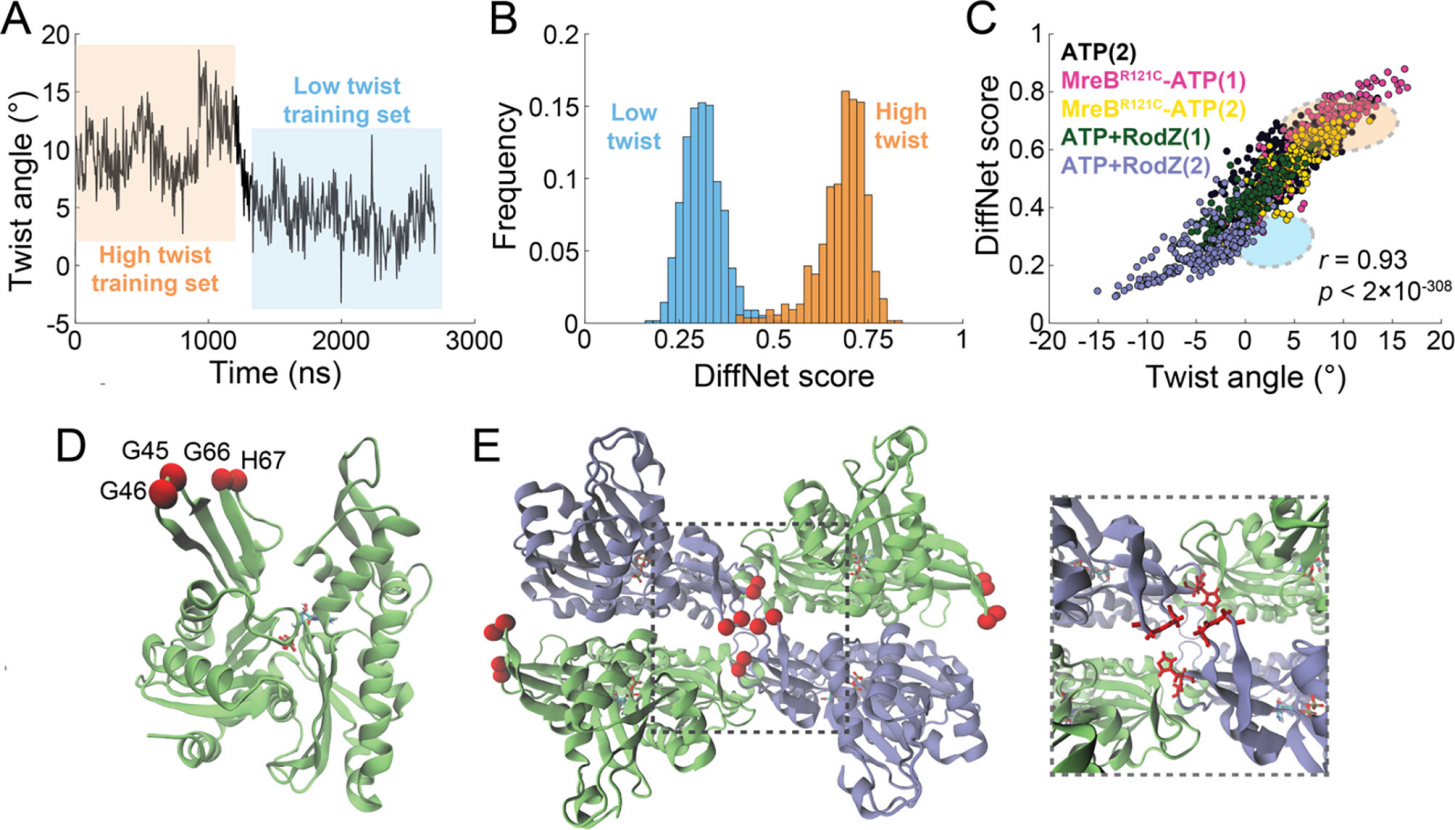
Deep learning identifies key residues responsible for predicting twist angle. A)   Training data used by DiffNets that defines high-twist (orange) and low-twist (blue) states. The trajectory is the 2.7-ms simulation of an ATP-bound double protofilament shown in Fig. 1C, left. The middle 2x2 doublet of the full 4x2 double protofilament was used for training. B)   DiffNet training scores for twist classification. High (orange) and low (blue) twist distributions are from the labeling of each simulation frame used during training. C)   Scoring of MreB MD simulations using the ATP twist-based DiffNet was highly correlated with twist angle. Each circle represents the frame from a simulation in a color labeling simulation and replicate number. Colored ellipses represent the training distributions for high (orange) and low (blue) twist states in (A,B). ATP(2): second replicate of ATP-bound double protofilament from Fig. 1C, right; R121C-ATP(1,2): replicates of MreB^R121C^ mutant bound to ATP; ATP+RodZ(1,2): ATP- and RodZ-bound double protofilament replicates. D)   MreB monomer structure with a-carbons of key residues identified from DiffNets based on twist classification shown as red spheres. Residues G45 and G46 were identified by training a twist-based DiffNet using only the middle doublet; residues G66 and H67 were identified by training a twist-based DiffNet using the full 4x2 double protofilament (Fig. S5). E)   Key residues in twist state classification are located at filament interfaces. Left: in the MreB middle doublet, key residues from (D) are located at the interface within a single filament (between green and blue subunits) and across antiparallel filaments (between the two blue subunits). Right: zoomed-in view of interface highlighting the key residues (red sticks).

**Fig. 4:**
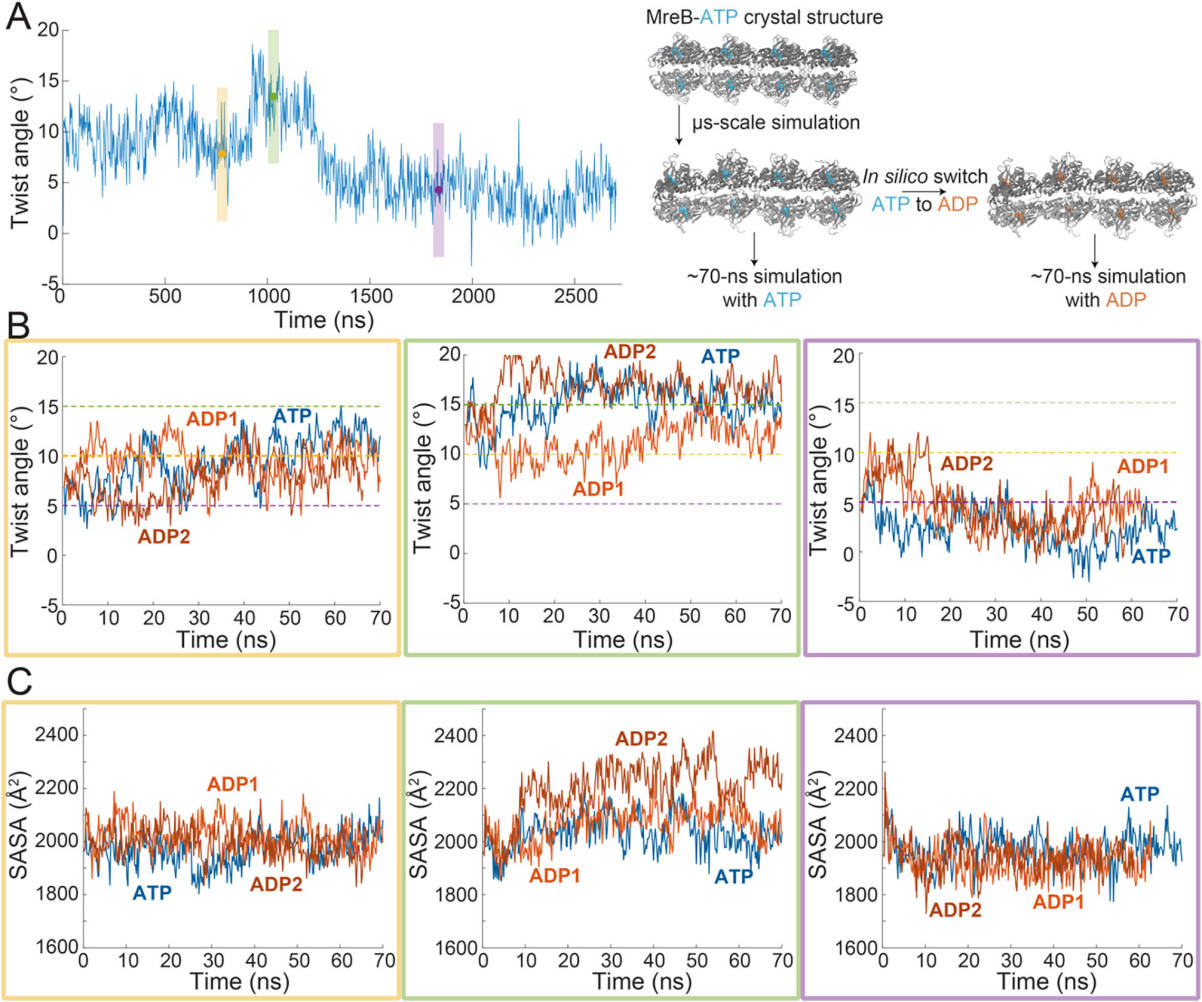
Nucleotide replacement typically does not alter twist angle. A)   The ATP was replaced with ADP to mimic nucleotide hydrolysis in structures sampled from an ATP-bound double protofilament at time points representing various twist states (purple = low; yellow = intermediate; green = high). Left: twist angle trajectory of an ATP-bound MreB double protofilament simulation with twist states labeled (colored dots are the frames from which structures were extracted). Right: each ATP-bound double protofilament structure was modified by replacing ATP with ADP and simulated for another ∼70 ns. B)   Twist angle trajectories for each nucleotide-replacement simulation, started from three twist states. Each replacement simulation is labeled with a replicate number, and colored bounding boxes correspond to twist states extracted from (A). Colored, dotted lines represent the three twist states (low, intermediate, and high). Left: trajectories starting from the intermediate twist state. Middle: trajectories starting from the high twist state. Right: trajectories starting from the low twist state. C)   Buried solvent accessible surface area (bSASA) is maintained for the double protofilament in each nucleotide-replacement simulation. Colored bounding boxes correspond to twist states extracted from (A). Left: trajectories starting from the intermediate twist state. Middle: trajectories starting from the high twist state. Right: trajectories starting from the low twist state.

To further interrogate the right-handed twisting of the RodZ simulation (Fig. 2A), we extracted a conformation with high right-handed twist and restarted short (<200 ns) simulations with and without RodZ bound. In the two simulations of ADP-bound filaments without RodZ, the right-handed twist quickly decreased to zero within tens of ns (Fig. S5A). Simulations of ATP-bound filaments with RodZ also exhibited somewhat reduced twist, despite relaxing more slowly (Fig. S5A). Interestingly, in the two ATP-bound simulations without RodZ, one untwisted within ∼ 50 ns, similar to ADP-bound simulations, while the other remained with high right-handed twist for 200 ns (Fig. S5A). In all simulations, bSASA values were high throughout (Fig. S5B), indicating that the double protofilament remained stable despite the large changes in twist. Thus, while MreB double protofilaments did not attain a conformation with right-handed twist in our µs-scale equilibrium simulations, the states adopted in RodZ-bound simulations can remain stable over hundreds of ns when RodZ is removed. It remains to be determined whether right-handed twist is inaccessible or accessible at low relative occupancy in equilibrium simulations.

To test whether the mechanical properties of the double protofilament depended on twist angle, we computed the twist modulus from the magnitude of the angular fluctuations (Methods) in each Steppi state across all simulations. The twist modulus across all states was approximately normally distributed with a relatively narrow spread (Fig. 2E, top), and there was no obvious bias of twist modulus with respect to either state lifetime or twist angle (Fig. 2E, bottom). Taken together, these data suggest that MreB double protofilaments can adjust twist angle without mechanical destabilization, supporting the hypothesis that each twist angle is directly tied to a particular quasi-stable state.

### 2.3 Deep learning predicts that twist angle is broadly correlated with the structural determinants that classify twist states

Thus far, our analysis has been focused on twist angle based on the apparent state transitions in simulations (Fig. 1,2). However, there are other structural properties that could also influence double protofilament conformation such as bending [43], making it unclear whether two conformations with the same twist angle actually represent a common state. To avoid assumptions about relevant structural features, we applied DiffNets, a deep-learning framework that leverages biochemical information about protein structural variants to learn structural contributions to functional behavior [56]. DiffNets has been successfully applied to detect subtle differences, for example beta-lactamase variant stabilities and myosin motor isoforms [56]. Here, we sought to learn which conformational features across MreB filaments contribute to twist.

We trained a DiffNets network (DiffNet) on the initial interval of an ATP-bound MreB simulation (Fig. 1C, left) with stable twist of ∼10.4° (Fig. 3A, orange) compared with an interval near the end of the same simulation with stable twist of ∼4.2° (Fig. 3A, blue). If the high (10.4°) and low (4.2°) twist states represent distinct conformational states, as assumed for the purpose of DiffNets input, the network should be able to predict twist-state dynamics in other simulations. We trained the DiffNet using only the middle doublet (Fig. 2B), since it was used for calculating the twist state. DiffNet scores for the structures in the two intervals displayed essentially non-overlapping distributions, with high score representing structures with high twist angle (Fig. 3B). When we applied the DiffNet to the other simulation of an ATP-bound free (no membrane) double protofilament, DiffNet scores were highly correlated with twist angle despite the large differences in twist angle distributions relative to the simulations in the training set (Fig. 3C, black). These results indicate that there is a set of structural features in common between high and low twist states in free ATP-bound filaments.

To determine if those structural features are conserved across other ATP-bound MreB double protofilament systems, we used the DiffNet to score simulations of MreB bound to RodZ or with the MreB^R121C^ mutation. In each case, DiffNet score was highly correlated with twist angle (Fig. 3C), consistent with our previous conclusion that these perturbations are shifting the occupancy of each state rather than altering the states themselves. Moreover, the portions of RodZ-bound MreB trajectories with negative (right-handed) twist exhibited even lower DiffNet scores than the training data (Fig. 3C, blue); likewise, very high twist angles from a R121C trajectory exhibited scores higher than training data (Fig. 3C, pink). Thus, the trained DiffNet can extrapolate to twist states outside the range of the training data. However, when we applied the network to an ADP-bound MreB simulation with stable twist angle ∼0.5° (Fig. 1D, left), scores were intermediate between the low and high twist intervals of the ATP-bound simulation (Fig. S6A), indicating that another factor disrupted predictions for ADP-bound simulations. Simulations of MreB bound to a membrane patch were scored higher than would be predicted from their twist angle alone, even though DiffNet scores correlated with twist angle (Fig. S6B,C).

To compare nucleotide-bound states, we trained a DiffNet to distinguish all time points in an ATP-bound MreB simulation (Fig. 1C, left) from all time points in an ADP-bound MreB simulation (Fig. 1D, right), both of which exhibited multiple twist angle transitions (Fig. S7A). Despite the overlap in twist angles between the two simulations (Fig. S7A), DiffNet scores were well separated between the two groups, with high scores corresponding to the ATP-bound simulation (Fig. S7B,C). However, application of the network to the other ADP-bound simulation, which had uniformly low twist angle (Fig. 1D, left), resulted in intermediate DiffNet scores (Fig. S7D), indicating that the network does not robustly separate states based only on nucleotide binding. Furthermore, ADP- and ATP-bound MreB filaments bound to membrane were all scored similarly as ATP filaments (Fig. S7E). Thus, we focused our structural analyses on the twist-based DiffNet trained on two states of an ATP-bound MreB double protofilament.

The DiffNet trained on the high and low twist states of the middle doublet of the ATP-bound simulation identified key residues whose movements contributed the most to classification, as scored by the top 50 interactions generated by DiffNets. These residues included G45 and G46, each of which is located within the exposed flexible loops in region IB (Fig. 3D, 1A); G46 was present in 34 of the top 50 interactions. To determine whether focusing on the middle doublet affected DiffNets classification, we trained another DiffNet on the high and low twist states of the entire 4x2 ATP-bound double protofilament. Scores from this DiffNet were also well separated between the two twist states (Fig. S8). Moreover, the key residues for classification included R63, P65, G66, and H67, all of which are located close to G45 and G46 in region IB (Fig. 3D, 1A); G66 and H67 were present in 39 of the top 50 interactions. In a double protofilament, all these residues are located at the filament interface (Fig. 3E), suggesting that our DiffNet indeed captured large-scale movement features related to filament twist.

Since individual residue movements can predict twist state, we wondered whether a DiffNet trained on individual subunits from the MreB double protofilament could achieve similar success in twist-state classification. We trained a DiffNet based solely on atomic coordinates from the P4 subunit (Fig. S9), which resides in the middle 2x2 doublet, with the same trajectories used to train the 4x2 and 2x2 DiffNets (Fig. 3A). Training was generally successful in classifying low and high twist states (Fig. S9A), although there was some overlap in the distribution of scores due to a decrease in scores of the high-twist state in later frames (Fig. S9B). Ultimately, this DiffNet failed to predict twist states from other simulations, instead identifying a negative correlation between score and twist angle (Fig. S9C). Among the residue interactions that contributed to training of the subunit-based DiffNet, there were fewer key residues at the filament interface. The top residue, H51, which resides in the IB subdomain, was found in 14 of the top 50 residue interactions, while the key twist residue G66 from the full filament was found only once (residues G45, G46, and H67 were not present in the top 50) (Fig. S9D). The infrequent presence of G45, G46, G66 and H67 in the subunit-based DiffNet is consistent with their localization at the filament interface (Fig. 3E). Thus, twist states are likely determined by large-scale movements along filament interfaces rather than specific internal rearrangements of individual subunits.

Interestingly, a large library of *E. coli* MreB mutants generated by selecting for changes in cell width [42] includes G47C (G45 in CcMreB) and G68C (G66 in CcMreB), both of which exhibited increased cell width of ∼1.3 µm compared with 0.97 µm for wild type. To determine the effect of these mutations on filament dynamics, we performed short (<200 ns) simulations of ATP-bound CcMreB double protofilaments with either the G45C or G66C mutation, as well as a wild-type simulation as a control (Fig. S10, Methods). While one of the G45C replicates quickly adopted a high twist above 10°, in agreement with wild-type filament behavior, the other replicate adopted only low twist angles below 5° (Fig. S10A). Similarly, simulations of G66C filaments displayed high twist in only one replicate (Fig. S10B). Since G45 and G66 are predicted by DiffNets to be important for determining twist state (Fig. 3D), we used our twist DiffNet (Fig. 3A,B) to score these mutant simulations. DiffNet score was highly correlated with twist angle in G45C filaments, with a relationship that overlapped with that of the control simulation (Fig. S10C) and in agreement with classification of other ATP-bound filaments (Fig. 3C). DiffNet scores of G66C were also correlated with twist angle, albeit with slightly lower DiffNet score for a given twist angle than other ATP-bound filament simulations (Fig. S10C). The bSASA was lower in both G66C simulations and the low-twist G45C replicate compared with that of wild-type simulations (Fig. S10D), indicating that the G45C and G66C mutations lower filament stability. Thus, the G45C and G66C mutations bias filaments toward lower twist states and compromise filament stability.

### 2.4 Twist states largely remain stable after nucleotide hydrolysis

Since simulations of ATP-bound MreB double protofilaments exhibited overall larger twist angles compared to ADP-bound filaments (Fig. 1C,D), we wondered whether high-twist ATP-bound states would transition to low-twist states after ATP was replaced by ADP, mimicking a hydrolysis event. To address this question, we selected three time points in simulations of ATP-bound filaments (Fig. 1C) at which the filament exhibited low, medium, or high twist angles (Fig. 4A, left) and either swapped the bound nucleotide to ADP to mimic hydrolysis, or maintained the ATP. We then carried out replicate simulations based on each of these structures for ∼70 ns (Fig. 4A, right). In the simulations with ATP, the filaments maintained their twist state regardless of the initial twist angle (Fig. 4B, blue curves), consistent with our previous interpretations that the twist angles observed in ATP-bound simulations are all relatively stable over tens to hundreds of nanoseconds.

When the binding nucleotide was switched to ADP, the twist angle was also stable for simulations initialized with low or medium twist angles (Fig. 4B, left and right, respectively). By contrast, in the ADP-bound simulations starting at ∼14° twist, only one of the replicates maintained the high-twist conformation, while the twist angle in the other replicate quickly decreased to and then equilibrated at an intermediate value (Fig. 4B, middle). In all simulations, regardless of the bound nucleotide, the bSASA between the middle doublet pairs was approximately constant (Fig. 4C), suggesting that each filament conformation remained stable.

To further characterize these simulations mimicking hydrolysis, we used the DiffNets trained on either distinct twist (Fig. 3A,B) or nucleotide-bound (Fig. S7A-C) states to score each frame. The nucleotide-based DiffNet failed to identify differences between simulation trajectories in which ATP was replaced by ADP (Fig. S11A), indicating that the MreB filaments in the two simulations likely remain in an ATP-like state and yet can still transition to a state with lower twist angle. The twist-based DiffNet successfully classified each twist state regardless of nucleotide (Fig. S11B), in agreement with our µs-scale Anton2 simulations (Fig. 3C). Thus, DiffNets does not distinguish between these ADP- and ATP-bound simulations, suggesting that MreB twist states are resilient upon nucleotide replacement on short time scales.

## 3 Discussion

Many bacterial cytoskeletal proteins are involved in cellular processes that span multiple length scales, such as the regulation of cell wall synthesis by MreB or of cell division by the tubulin homolog FtsZ [5], [11]. As a result, the filaments must reorganize as the cell develops [17], and the ability to transition between states may play a major role in such spatial reorganization. How does a filament adopt multiple states? One possibility is that the subunits possess multiple binding interfaces, which has been hypothesized as the mechanism by which GTP hydrolysis alters the bending of FtsZ protofilaments without affecting subunit conformation [19]. A mutation within a helix of *Staphylococcus aureus* FtsZ can alter the inter-subunit binding interface and introduce protofilament twist, which leads to altered patterns of cell-wall insertion along the FtsZ helix and a transition from coccoid growth to elongation [37]. For *T. maritima* MreB, protofilament bending appears to be cooperative [51], a behavior that has been linked to intrasubunit conformational changes [8] and may also be connected with binding to the membrane [43] or to RodZ [9], [35], [53].

While simulations from our previous study showed that ADP-bound filaments adopt lower twist angles than ATP-bound filaments [43], the μs-scale simulations in this study demonstrate that both nucleotide-bound configurations are capable of accessing low and high twist angles. Our analysis suggests that the nucleotide may bias twist-state distributions toward either high (ATP-like) or low (ADP-like) twist (Fig. 2). Furthermore, in simulations where ATP was replaced with ADP, twist angles mostly remained stable (Fig. 4), indicating that structural factors independent of nucleotide hydrolysis are at least partially responsible for twist-state transitions.

Based on our findings, we hypothesize that intersubunit interactions involving flexible loops in the IB subdomain are crucial for determining twist state (Fig. 3D,E). Simulations of filaments with G45C or G66C (mutations that increase cell width *E. coli* [42]) displayed a bias toward lower twist angles (Fig. S10A,B) and lower stability (Fig. S10D). Compromised filament stability may generally reduce MreB function and its interactions with binding partners. Future MD simulations focusing on these interactions may reveal the mechanism of twist-state transitions, and deep scanning mutagenesis [2] of MreB will enable experimental validation of the links between cellular phenotypes and the functional consequences of interfacial disruption.

The ability of a cytoskeletal filament to adopt multiple states can provide several advantages. MreB plays a central role in cell shape and size determination, which are important determinants of cellular physiology [7], [58]. Cell size and width vary with growth rate across nutrient conditions [39], [49], and thus the pattern of MreB-directed cell wall synthesis [48] must adjust. Since twist angle is linked to MreB patterning [43] and MreB curvature preference varies as a function of cell width [42], being able to adopt multiple twist states may enable MreB to adapt to cell width. The size of double protofilaments that can be explored in MD simulations over long time scales is currently limited, and double protofilaments with more subunits may have a different twist landscape than the 4x2 systems that we have focused on in this study. In that case, since double protofilament length is likely dependent on MreB concentration, modulation of the twist landscape along with transcriptional feedback on *mreB* expression as a function of cell width [44] could enable homeostasis of cell width.

MD simulations of bacterial cytoskeletal filaments on the 100-ns time scale [8], [19] have predicted conformational changes that were subsequently validated experimentally [28], [52], and have provided estimates of filament mechanical properties that can be used to predict intracellular organization [43]. However, such simulations have also revealed state transitions rather than complete equilibration [19], and this study shows that longer, µs-scale simulations may be critical to understand the full spectrum of possible conformations. Like MreB, prokaryotic actin homologs such as FtsA, ParM, and crenactin display nucleotide- and filament-dependent subunit changes [36], suggesting that their filaments may also have multiple quasi-stable states. A combination of specialized supercomputers such as Anton2 with computational strategies such as steered or accelerated MD will enable comprehensive exploration of the state landscape, and future studies should focus on quantifying the occupancy of each state. Moreover, deep learning algorithms such as DiffNets can highlight the specific residues responsible for state differences, facilitating targeted mutagenesis experiments and MD simulations of mutants. Ultimately, a comparison of behaviors among cytoskeletal proteins may reveal fundamental rules that couple filament structure and function.

## 4 Methods

### 4.1 Equilibrium MD simulations

All µs-scale simulations were performed on the Anton2 supercomputer [40]; ∼100 ns-scale simulations were performed using the MD package NAMD [38]. Simulations were set up as previously described [43]. Briefly, the CHARMM36 force field [4] and CMAP corrections [30] were used, with water molecules described with the TIP3P model [23]. Integration time step was 2 fs [47]. Bonded terms and short-range, non-bonded terms were evaluated every time step, and long-range electrostatics were evaluated every other time step. Setup, analysis, and rendering of the simulation systems were performed with VMD [20].

### 4.2 Simulated systems

MD simulations performed in this study are described in Table S1. Unless otherwise noted, systems were initialized using equilibrated simulations from [43]. The bound nucleotide was replaced by ATP or ADP with chelating Mg^2+^ ions for all simulated systems, a bounding water box was added to surround the complex, and sodium or chloride ions were added to neutralize the simulated system. Mutations G45C and G66C were created in VMD using the *mutate* tool [20].

### 4.3 Membrane binding

MD systems with a membrane were generated as previously described [43]. Briefly, the membrane plugin in VMD was used to add an all-atom patch of palmitoyloleoyl phosphatidylethanolamine (POPE) molecules, neutralized by NaCl, to an equilibrated 4x2 filament MreB simulation. The filament was placed approximately 10 Å from the membrane patch and allowed to freely interact during a 120 ns simulation. This final state was used as the initial system in Anton2 simulations.

### 4.4 Calculation of buried solvent-accessible surface area (SASA)

The buried SASA between two molecules was calculated from three quantities: the SASA of each molecule by itself (denoted as *A*_1_ and *A*_2_), and the SASA of the complex of the two molecules when interacting (denoted as *A*_1+2_). The buried SASA between the two molecules is

buried SASA = (*A*_1_ + *A*_2_−*A*_1+2_)/2.

For each molecule or molecule complex, its corresponding SASA was calculated in VMD using the command *measure sasa*, with 1.40 Å as the van der Waal’s radius for water molecules.

### 4.5 Calculation of filament bending and twisting angles

For each simulation frame, a local coordinate system was defined by three unit vectors (**d_1_**, **d_2_**, **d_3_**) as previously described [19]. In the MreB filament, **d_3_** is largely parallel to the filament, **d_2_** is approximately perpendicular to the membrane plane, and **d_1_** = **d_3_** × **d_2_**. Twist angle is the rotation angle around **d_3_**.

### 4.6 Steppi analyses

State-change identification was performed using Steppi [27], [57], a change-point analysis package written in MATLAB. The algorithm assumes features of the noise in each state and builds a model that identifies transitions between discrete states. Parameters for state-change identification in MreB twist trajectories were chosen for a one-dimensional process as follows: state level μ = unfixed, level slope α = 0, nearest-neighbor coupling ε = 0.6, noise “stiffness” *k* = 0.2. These parameters were chosen to optimize state identification in agreement with initial clustering analyses.

### 4.7 *K*-means clustering of Steppi-identified states

Twist-state clustering was performed with the *kmeans* function in MATLAB. A four-cluster partitioning of the data was based on the distribution of all twist states identified by Steppi. The clustering was performed separately on the set of free (no membrane, no RodZ) ADP- and ATP-bound filament simulations and on the set of all simulation trajectories.

### 4.8 Calculation of twist modulus

Twist modulus was calculated as previously described [43]. Briefly, a Gaussian function was fit to the distribution of twist angles in each state, and the standard deviation, σ, was used in a small-angle approximation to calculate the twist modulus as $K=k_{\text{B}}T\frac{l}{\sigma^{2}}$, where l≈5nm is the size of an MreB monomer. Note that any slow variation in the average twist angle will lead to an overestimation of σ and hence an underestimation of *K*.

### 4.9 DiffNets analyses

Inputs for DiffNet training were prepared according to [56] as follows: (1) NAMD trajectories from Anton2 simulations were processed using VMD [20] to extract only the relevant residue coordinates; (2) trajectories were converted from *dcd* format to *xtc* using the *mdconvert* command-line script from the MDTraj python package [31]; (3) trajectories were pre-processed using the whitening procedure detailed in the DiffNets package; (4) each DiffNet was trained using identical hyper-parameters (10 epochs, 50-fold dimensional reduction, 32-frame batch size, *nnutils.sae* architecture); (5) DiffNet clustering analysis was performed to identify key residue interactions, of which the top 50 were chosen for further analysis.

All trajectories used for DiffNet training and prediction were performed on residue alpha-carbon atoms. Full (4x2) and doublet (2x2) filament trajectories were further simplified by removing residues 1 to 13, which comprise the flexible *N*-terminal section added to PDB: 4CZF in previous simulations [43]. Subunit-based DiffNets were trained on trajectories after removing residues 335 to 347 (the flexible C-terminus), in addition to residues 1 to 13. These deletions reduced unwanted residue-score interactions during training.

## Declaration of Competing Interest

The authors declare the following financial interests/personal relationships which may be considered as potential competing interests: Kerwyn Casey Huang reports financial support was provided by National Science Foundation. Kerwyn Casey Huang reports financial support was provided by Chan Zuckerberg Biohub. Handuo Shi reports financial support was provided by James S McDonnell Foundation.
