# Supplementary material for: Multiple conserved states characterize the twist landscape of the bacterial actin homolog MreB
Source: Comput Struct Biotechnol J. 2022 Oct 7;20:5838–46. doi: 10.1016/j.csbj.2022.10.008 (PMC9627593; doi:10.1016/j.csbj.2022.10.008)
Supplement: Supplementary data 1 [file mmc1.docx]

**Supplemental Information for “Multiple conserved states characterize the twist landscape of the bacterial actin homolog MreB”**

**Authors:** Benjamin D. Knapp^1^, Michael D. Ward^2,3^, Gregory R. Bowman^2,3,4^, Handuo Shi^5,6,†^, Kerwyn Casey Huang^1,5,6,7,†^

**Affiliations:**

^1^Biophysics Program, Stanford University, Stanford, CA 94305, USA

^2^Department of Biochemistry & Molecular Biophysics, Washington University School of Medicine, St. Louis, MO 63130, USA

^3^Center for the Science and Engineering of Living Systems, Washington University in St. Louis, St. Louis, MO 63130, USA

^4^Department of Biochemistry and Biophysics, University of Pennsylvania, Philadelphia, PA 19104, USA

^5^Department of Microbiology and Immunology, Stanford University School of Medicine, Stanford, CA 94305, USA

^6^Department of Bioengineering, Stanford University, Stanford, CA 94305, USA

^7^Chan Zuckerberg Biohub, San Francisco, CA 94158, USA

^†^To whom correspondence should be addressed: [handuo@stanford.edu](mailto:handuo@stanford.edu), [kchuang@stanford.edu](mailto:kchuang@stanford.edu)

**Supplemental Tables**

**Table S1: List of MD simulation systems in this study.**

| **Name** | **Computing resource** | **Ligand** | **Atoms (×1000)** | **Time** | **Replicates** |
| --- | --- | --- | --- | --- | --- |
| 4x2 ATP | Anton2 | ATP and Mg^2+^ | 356 | 2.7 µs; 2 µs | 2 |
| 4x2 ADP | Anton2 | ADP and Mg^2+^ | 356 | 1 µs each | 2 |
| 4x2 ATP (R121C) | Anton2 | ATP and Mg^2+^ | 356 | 1 µs each | 2 |
| 4x2 ATP RodZ | Anton2 | ATP and Mg^2+^ | 481 | 1 µs each | 2 |
| 4x2 ATP membrane | Anton2 | ATP and Mg^2+^, membrane patch | 434 | 1 µs each | 2 |
| 4x2 ADP membrane | Anton2 | ADP and Mg^2+^, membrane patch | 434 | 1 µs each | 2 |
| 4x2 ATP (R121C) membrane | Anton2 | ATP and Mg^2+^, membrane patch | 434 | 2 µs; 1 µs | 2 |
| 4x2 ATP RodZ membrane | Anton2 | ATP and Mg^2+^, membrane patch | 482 | 2 µs; 1 µs | 2 |
| 4x2 ATP, high initial twist | XSEDE | ATP and Mg^2+^ | 356 | 70 ns | 1 |
| 4x2 ATP, intermediate initial twist | XSEDE | ATP and Mg^2+^ | 356 | 70 ns | 1 |
| 4x2 ATP, low initial twist | XSEDE | ATP and Mg^2+^ | 356 | 70 ns | 1 |
| 4x2 ADP, high initial twist | XSEDE | ADP and Mg^2+^ | 356 | 70 ns each | 2 |
| 4x2 ATP, intermediate initial twist | XSEDE | ADP and Mg^2+^ | 356 | 70 ns each | 2 |
| 4x2 ATP, low initial twist | XSEDE | ADP and Mg^2+^ | 356 | 70 ns each | 2 |
| 4x2 ATP, negative twist | XSEDE | ATP and Mg^2+^ | 356 | 125, 200 ns | 2 |
| 4x2 ADP, negative twist | XSEDE | ADP and Mg^2+^ | 356 | 125 ns each | 2 |
| 4x2 ATP +RodZ, negative twist | XSEDE | ATP and Mg^2+^ | 481 | 150 ns each | 2 |
| 4x2 ATP | XSEDE | ATP and Mg^2+^ | 292 | 100 ns | 1 |
| 4x2 ATP (G45C) | XSEDE | ATP and Mg^2+^ | 292 | 180 ns each | 2 |
| 4x2 ATP (G66C) | XSEDE | ATP and Mg^2+^ | 292 | 140, 175 ns | 2 |

**Supplemental Figures**

**
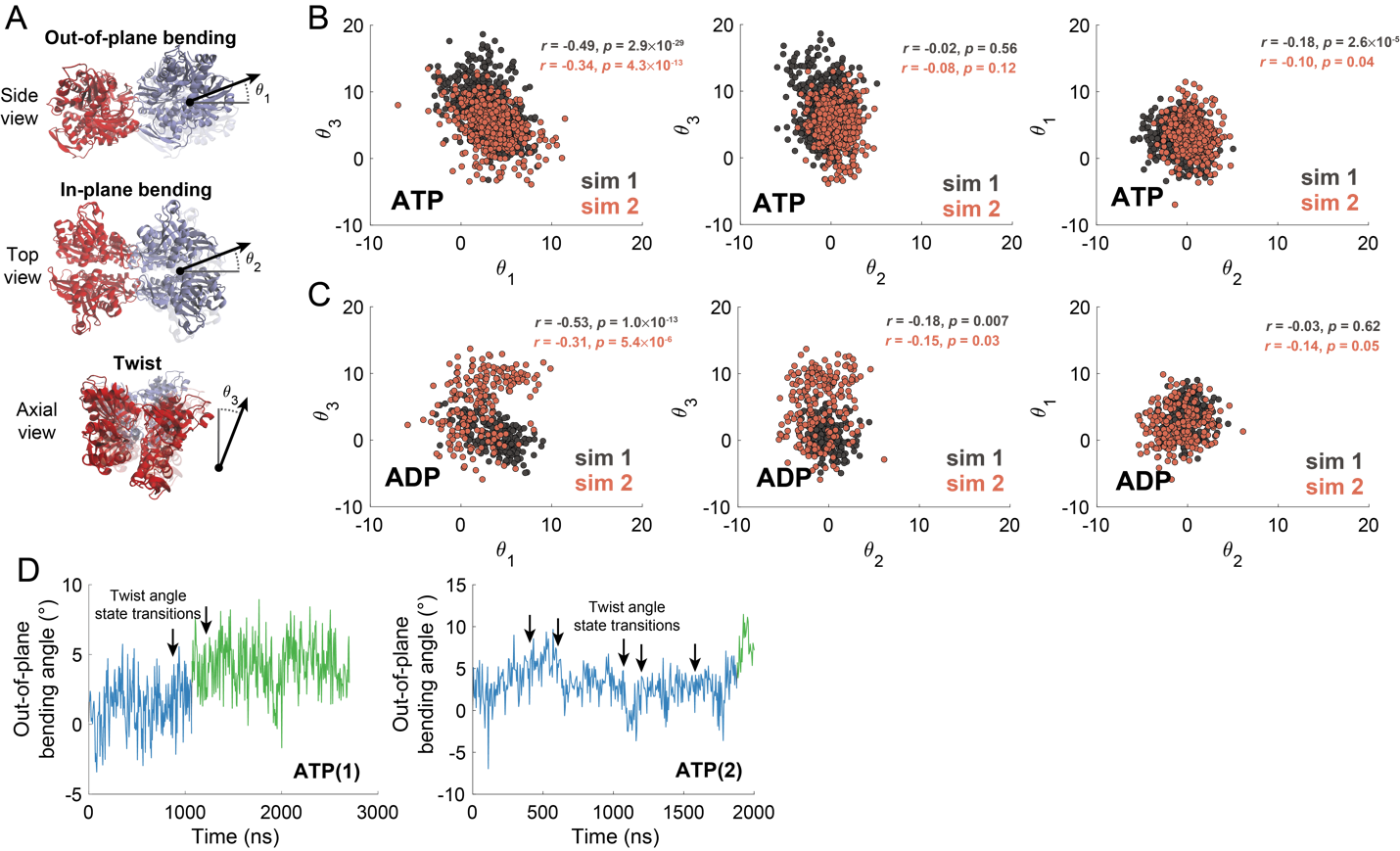
**

**Figure S1: MreB double protofilament behavior is dominated by twist dynamics.**

1. Definitions of bending and twist angles in the middle doublet of an MreB double protofilament. Antiparallel pairs are shown in red or blue. (Top) Side view of middle doublet showing out-of-plane bending angle *θ*_1_ (original orientation of blue pair is shown with transparency). (Middle) Top view of middle doublet showing in-plane bending angle *θ*_2_ (original orientation of blue pair is shown with transparency). (Bottom) Axial view (down the filament axis) of middle doublet showing twist angle *θ*_3_ (original orientation of red pair is shown with transparency).
2. Correlations between bending and twist angles of ATP-bound MreB double protofilaments. (Left) Correlation between twist angle (*θ*_3_) and out-of-plane bending angle (*θ*_1_) for each simulation replicate (black, red). (Middle) Correlation between twist angle (*θ*_3_) and in-plane bending angle (*θ*_2_) for each simulation replicate (black, red). (Right) Correlation between out-of-plane bending angle (*θ*_1_) and in-plane bending angle (*θ*_2_) for each simulation replicate (black, red). Only the correlation between *θ*_1_ and *θ*_3_ was consistently significant.
3. Correlations between bending and twist angles of ADP-bound MreB double protofilaments. (Left) Correlation between twist angle (*θ*_3_) and out-of-plane bending angle (*θ*_1_) for each simulation replicate (black, red). (Middle) Correlation between twist angle (*θ*_3_) and in-plane bending angle (*θ*_2_) for each simulation replicate (black, red). (Right) Correlation between out-of-plane bending angle (*θ*_1_) and in-plane bending angle (*θ*_2_) for each simulation replicate (black, red). Only the correlation between *θ*_1_ and *θ*_3_ was consistently significant.
4. Steppi identification of state transitions in out-of-plane bending angle (*θ*_1_) of ATP-bound MreB double protofilaments. Each identified state is colored blue or green. Arrows indicate time points at which twist-angle transitions were identified by Steppi.


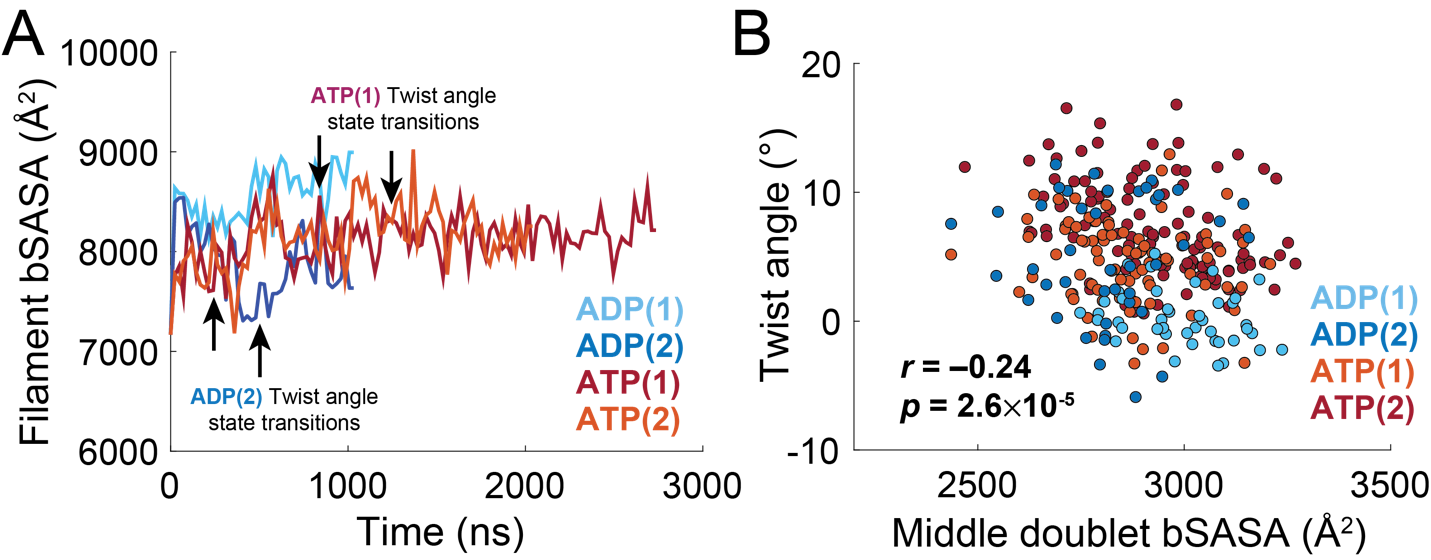


**Figure S2: MreB double protofilaments are stable during microsecond simulations and throughout twist transitions.**

1. Buried solvent-accessible surface area (bSASA) of ADP-bound (light blue, dark blue) and ATP-bound (orange, red) MreB double protofilament replicate simulations.
2. Twist angle and bSASA of the double protofilament middle doublet were weakly correlated (Fig. 1A,B). ADP-bound (light blue, dark blue) and ATP-bound (orange, red) replicate simulations are overlaid.

**
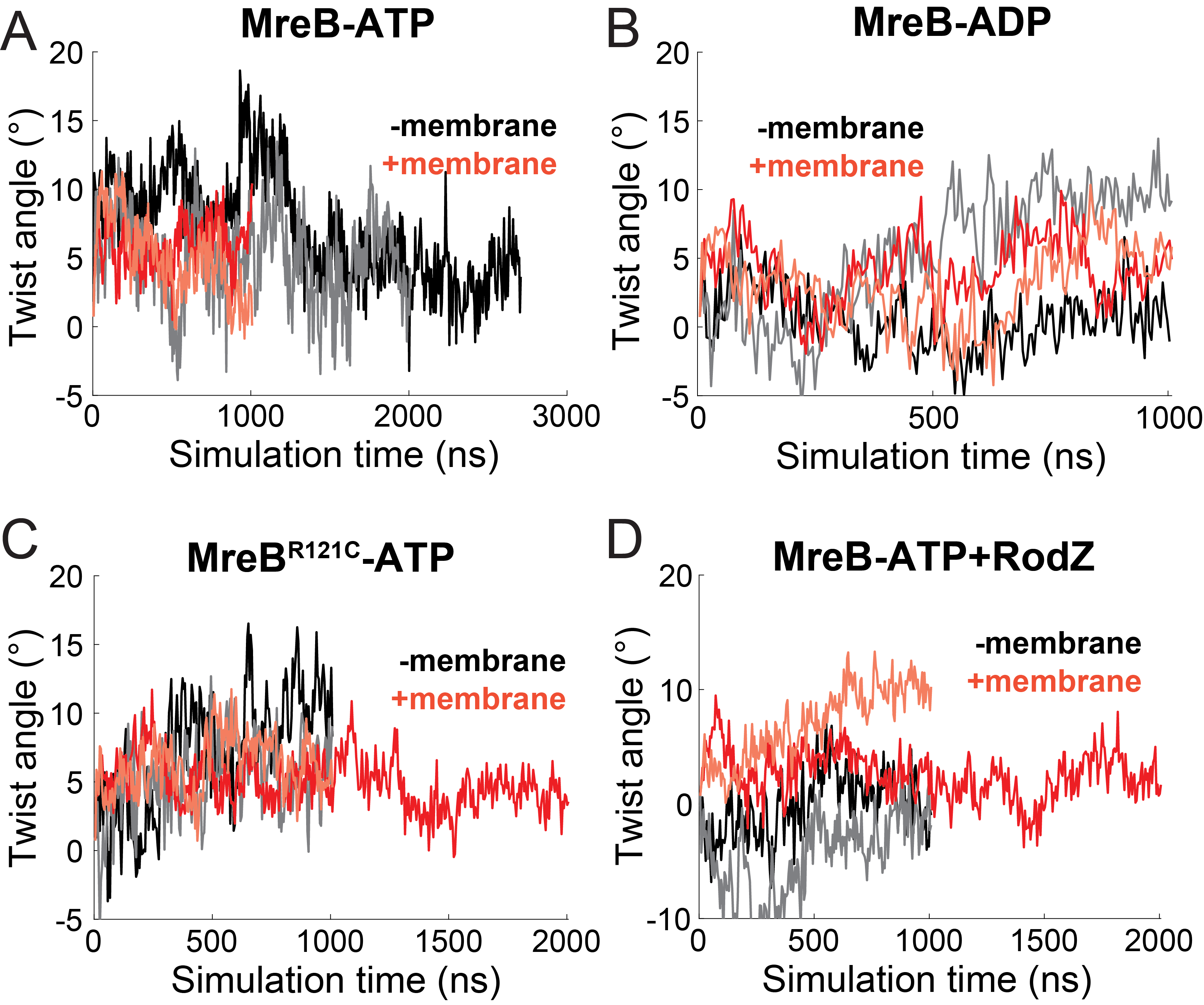
**

**Figure S3: Anton2 enables microsecond-scale simulations of MreB double protofilaments.**

1. Twist angles of ATP-bound MreB double protofilaments. Dark gray and black trajectories are replicate simulations of ATP-bound MreB in water only, and red and orange trajectories are replicate simulations of ATP-bound MreB bound to a membrane patch.
2. Twist angles of ADP-bound MreB double protofilaments. Dark gray and black trajectories are replicate simulations of ADP-bound MreB in water only, and red and orange trajectories are replicate simulations of ADP-bound MreB bound to a membrane patch.
3. Twist angles of ATP-bound MreB^R121C^ double protofilaments. Dark gray and black trajectories are replicate simulations of ATP-bound MreB^R121C^ in water only, and red and orange trajectories are replicate simulations of ATP-bound MreB^R121C^ bound to a membrane patch.
4. Twist angles of ATP- and RodZ-bound MreB double protofilaments. Dark gray and black trajectories are replicate simulations of ATP- and RodZ-bound MreB in water only, and red and orange trajectories are replicate simulations of ATP- and RodZ-bound MreB bound to a membrane patch.

**
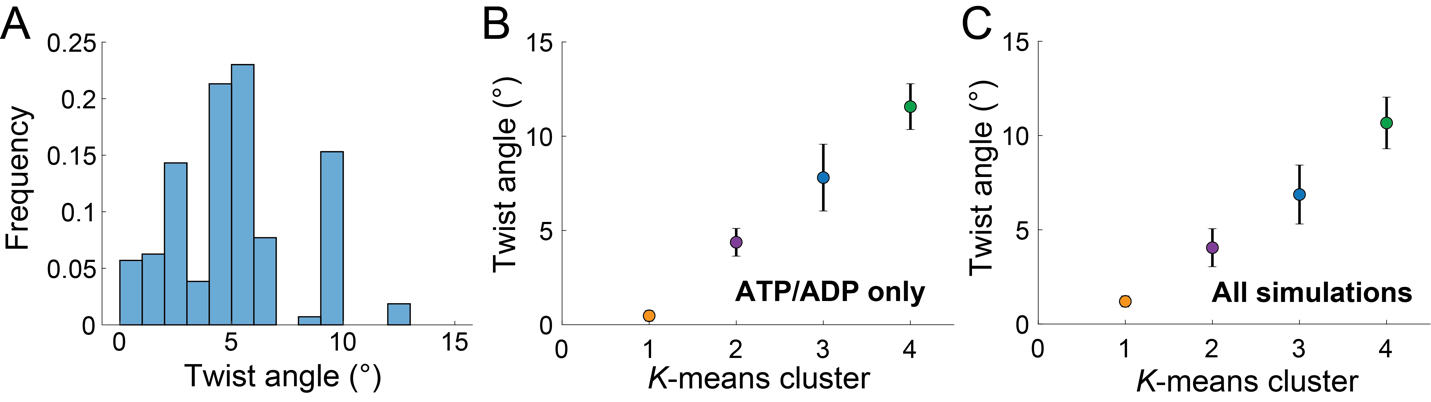
**

**Figure S4: Distribution of twist-state angles remains multimodal when weighted by twist-state lifetime.**

1. The peaks in the lifetime-weighted distribution overlap with those in Fig. 2B. The lifetime of twist states that were truncated by termination of the simulation was assumed to end at that point.
2. Average result of *K*-means clustering with *n*=4 of Steppi-identified twist states across ATP- and ADP-bound free (no membrane, no RodZ) double protofilament simulations. Solid circles with error bars represent the standard deviation of each cluster’s mean distribution after 1000 re-samplings.
3. Average result of *K*-means clustering with *n*=4 of Steppi-identified twist states across all simulations. Solid circles with error bars represent the standard deviation of each cluster’s mean distribution after 1000 re-samplings.

**
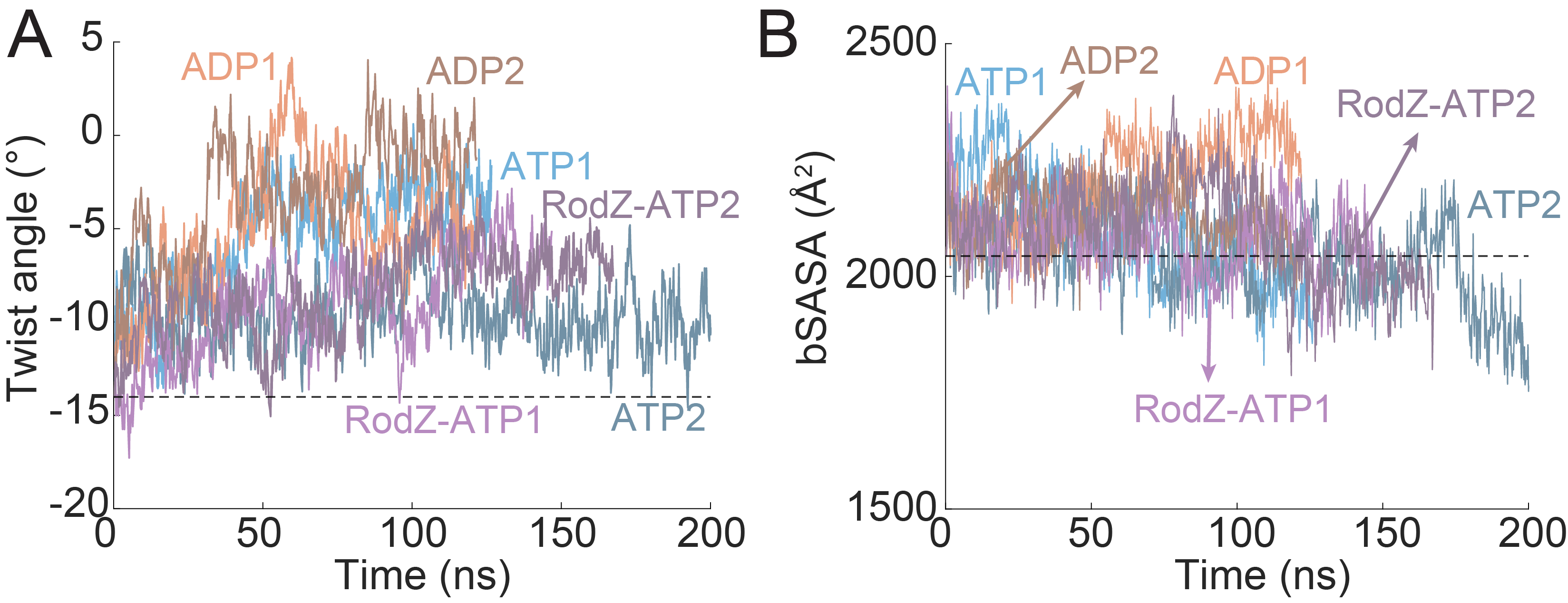
**

**Figure S5: ATP-bound MreB double protofilaments can stably adopt right-handed twist.**

1. Twist angle during simulations initialized using a conformation with right-handed (negative) twist from a simulation of RodZ-bound MreB. ADP-bound filaments (orange, brown), RodZ-bound ATP filaments (pink, purple), and ATP-bound filaments without RodZ (light blue, dark blue) are shown. Dashed line represents the initial twist angle.
2. Buried solvent-accessible surface area (bSASA) for each right-handed twist trajectory in (A). ADP-bound filaments (orange, brown), RodZ-bound ATP filaments (pink, purple), and ATP-bound filaments without RodZ (light blue, dark blue) are shown. Dashed line represents the initial bSASA. Arrows are visual aids for identifying overlapping trajectories.

**
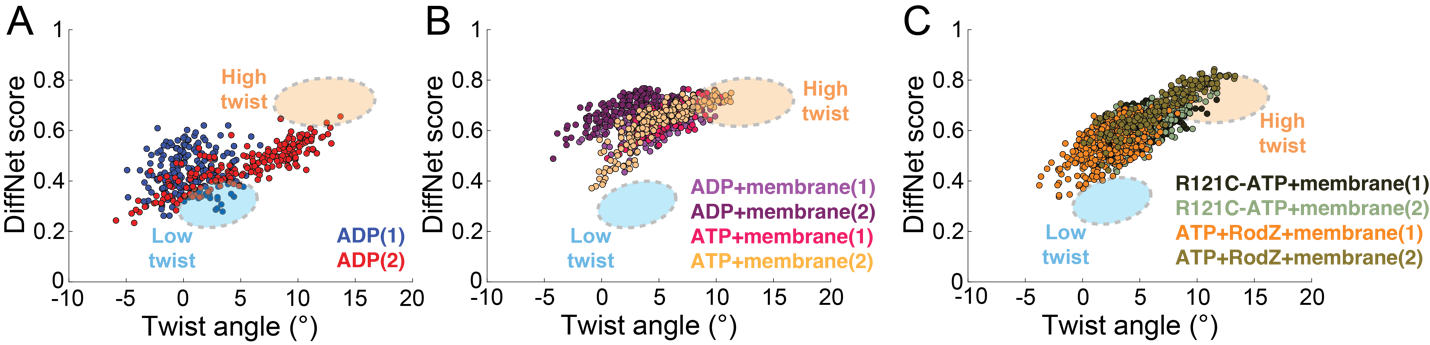
**

**Figure S6: DiffNet based on ATP-bound filament twist has moderate predictive success on ADP- and membrane-bound systems.** In (A-C), the DiffNet was trained on two twist states of a 2x2 ATP-bound doublet as in Fig. 3, and score distributions of high (orange) and low (blue) twist training data are shown as ellipses.

1. DiffNet scores were correlated with twist angle in only one simulation of an ADP-bound double protofilament (ADP(2)). Scores of each frame in replicate simulations (blue, red circles) are shown.
2. DiffNet scores were generally correlated with twist angle in simulations of ADP- and ATP-bound double protofilaments bound to a membrane patch. Scores of each frame in replicate simulations of ADP-bound (light purple, dark purple circles) and ATP-bound (pink, yellow circles) systems are shown.
3. DiffNet scores were generally correlated with twist angle in simulations of ATP-bound MreB^R121C^ and MreB+RodZ double protofilaments bound to a membrane patch. Scores of each frame in replicate simulations of MreB^R121C^ (black, light green circles) and MreB+RodZ (orange, olive green circles) systems are shown.

**
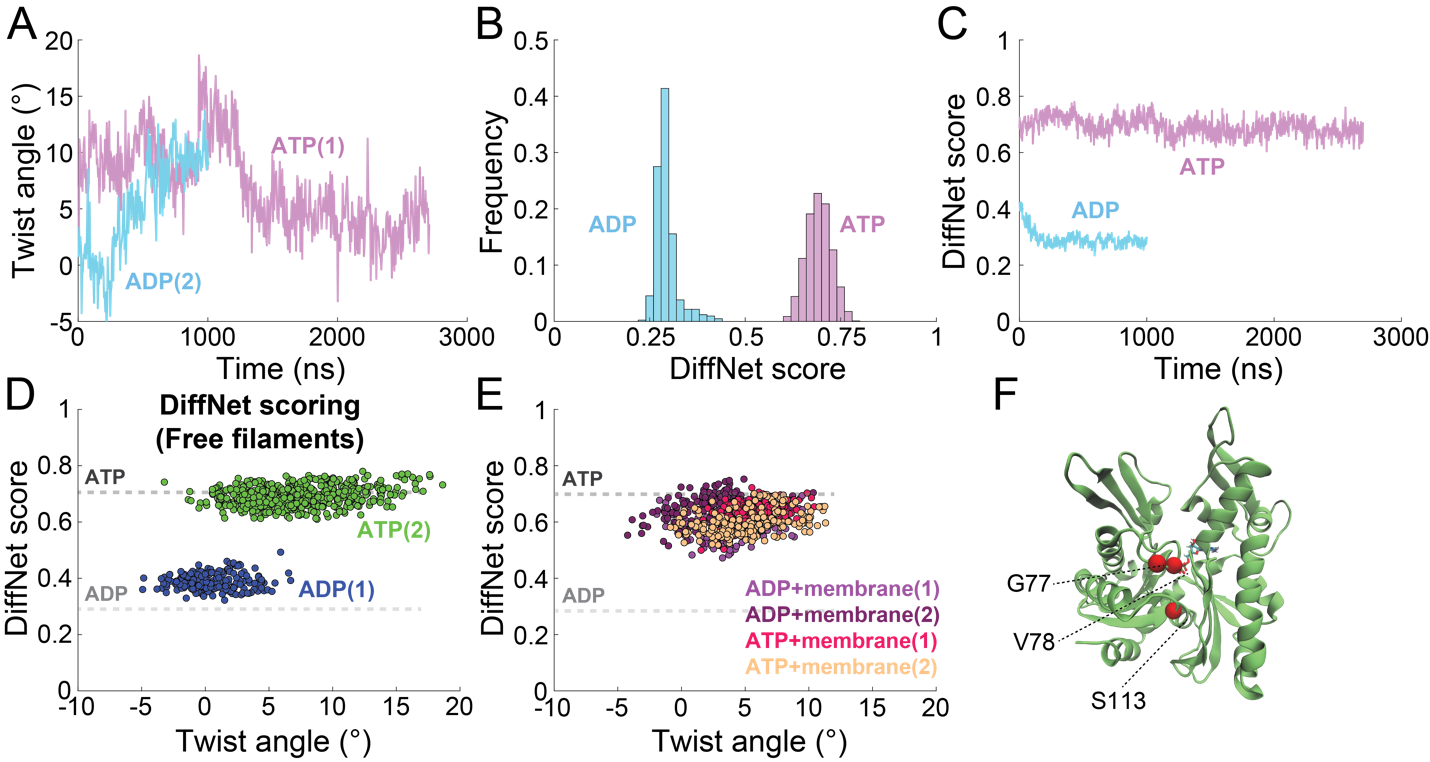
**

**Figure S7: Nucleotide-based DiffNet does not predict twist state of membrane-bound double protofilaments.**

1. The first ATP-bound (pink) and second ADP-bound (cyan) simulation in Fig. 1C and 1D, respectively, were used to train a DiffNet based on the middle 2x2 doublet due to their similar range of twist values.
2. The nucleotide-based DiffNet successfully classified training data. Shown are distributions of DiffNet scores for frames of the ATP-bound (pink) and ADP-bound (cyan) double protofilament simulations.
3. Nucleotide-based DiffNet scoring was stable across the simulation trajectories used for training, despite the variations in twist angle (A).
4. The nucleotide-based DiffNet successfully classified other simulations of free double protofilaments that were not used for training. Each circle represents a frame from a simulation of an ATP- (green) or ADP-bound (blue) system. Also shown are the mean labeling values from DiffNet training (B) for the ADP-bound (light gray dashed line, mean = 0.3) and ATP-bound (dark gray dashed line, mean = 0.7) filaments.
5. Nucleotide-based DiffNet was unable to predict the nucleotide of membrane-bound filaments. Shown are DiffNet scores for replicates of ADP-bound (light purple, dark purple) and ATP-bound (pink, yellow) double protofilaments bound to a membrane patch. Dashed lines represent the mean DiffNet score of the training data (B) for the ADP-bound (light gray, mean = 0.3) or ATP-bound (dark gray, mean = 0.7) double protofilaments.
6. Key residues identified by the nucleotide-based DiffNet are near the nucleotide-binding pocket. Shown is an MreB monomer (green) with key residues marked with red spheres. G77, V78, and S113 were present in 41 of 50 of the top residue-residue interactions that contributed to nucleotide-based DiffNet scoring.


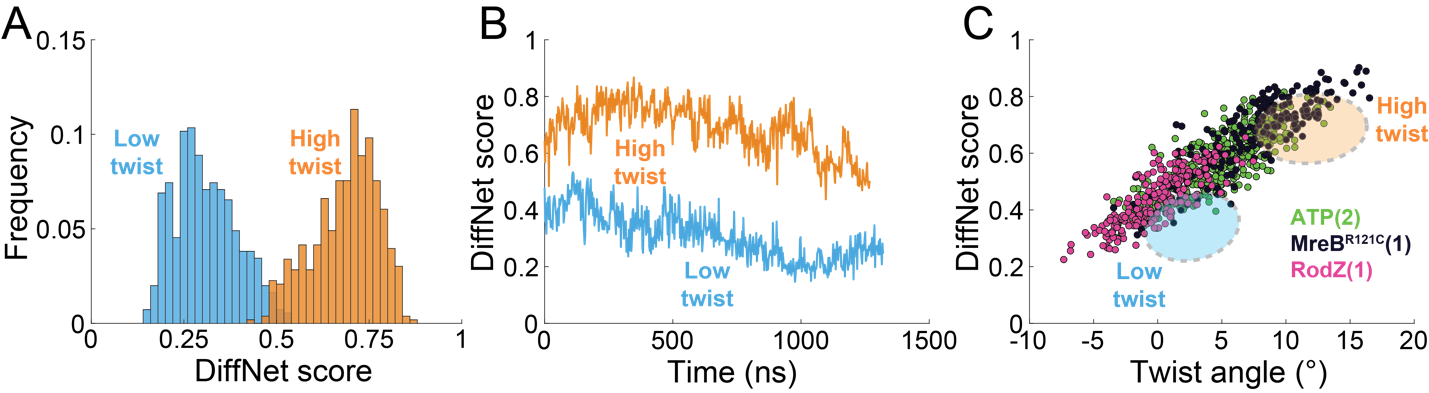


**Figure S8: Twist-based DiffNet trained on the full 4x2 double protofilament successfully predicts twist angle of other ATP-bound simulations.**

1. DiffNet trained on twist states from the first 4x2 double protofilament filament simulation in Fig. 1C successfully classified twist states from frames in the training data. Shown are the distributions of DiffNet scores of the high (orange) and low (blue) twist-state trajectories from Fig. 3A.
2. Twist-based 4x2 DiffNet scoring was reasonably stable over the trajectories used for training (A).
3. Twist-based 4x2 DiffNet scores were generally correlated with twist angle for ATP-bound double-protofilament simulations. Each circle represents a frame from one of the simulations in Fig. 3C, none of which were used for training. Colored ellipses represent the score distributions of the high (orange) and low (blue) twist states of the training data in (A,B).

**
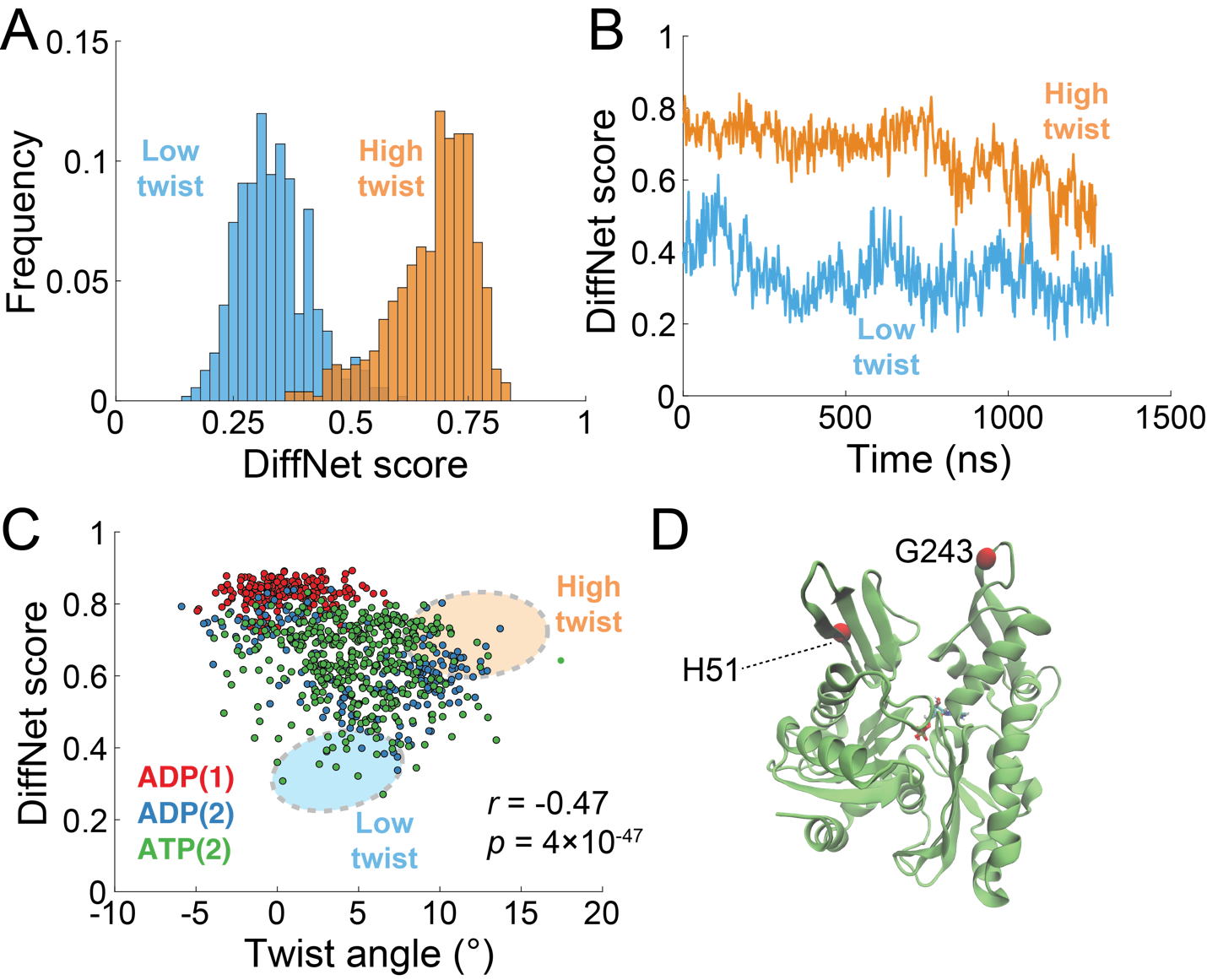
**

**Figure S9: Subunit-only twist-based DiffNet fails to predict twist states.**

1. A DiffNet trained using a single subunit bound to ATP (segment P4 within the double protofilament) was able to classify twist states reasonably successfully. Shown are the score distributions for each frame of the training data of high (orange) and low (blue) twist states.
2. Subunit-only twist-based DiffNet scoring was only somewhat stable over the trajectories used for training (Fig. 3A).
3. Subunit-only twist-based DiffNet scores were negatively correlated with twist angle in frames of other simulations not used for training, and did not agree with the score distributions (colored ellipses) of the high (orange) and low (blue) twist states of the training data in (A,B).
4. Key residues identified by the subunit-only twist-based DiffNet were distinct from those predicted by the twist-based DiffNet in Fig. 3D,E. Shown is an MreB monomer (green) with key residues marked with red spheres. H51 and G243 were present in 22 of the top 50 residue-residue interactions that contributed to subunit-only twist-based DiffNet scoring.


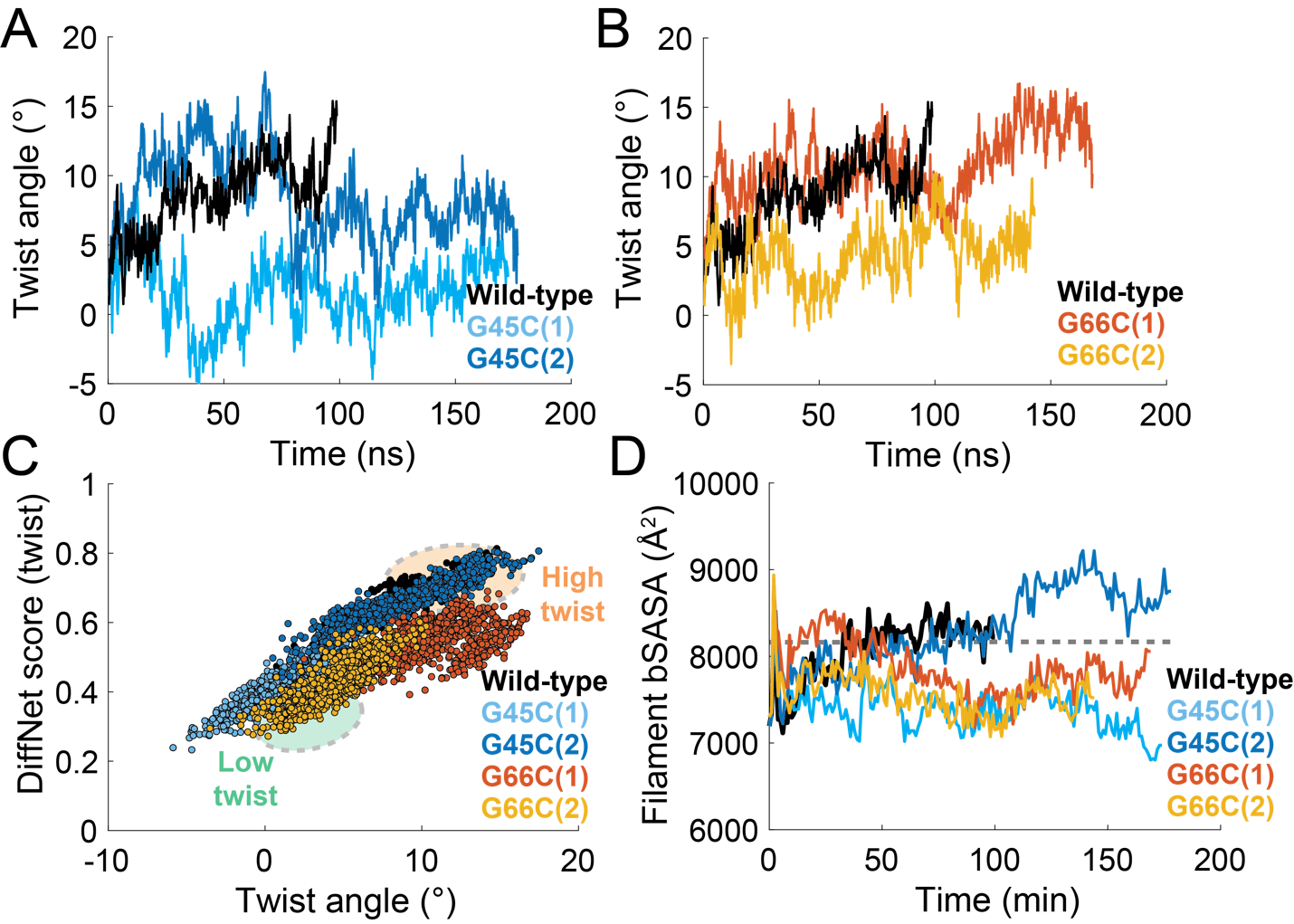


**Figure S10: Mutations of key twist residues identified by DiffNets result in lower twist angles and decreased filament stability.**

1. Twist angle during ATP-bound MreB^G45C^ double protofilament trajectories. Replicates are shown (light blue, dark blue) with wild-type simulation overlaid (black).
2. Twist angle during ATP-bound MreB^G66C^ double protofilament trajectories. Replicates are shown (gold, orange) with control simulation overlaid (black).
3. Scores of ATP-bound MreB^G45C^ and MreB^G66C^ trajectories predicted using the DiffNet trained on twist angles of an ATP-bound wild-type MreB trajectory (Fig. 3A,B) were highly correlated with trajectory twist angle. Each circle represents a frame from a simulation in a color labeling the simulation and replicate number. Colored ellipses represent the training distributions for high (orange) and low (green) twist states from Fig. 3.
4. Buried solvent-accessible surface area (bSASA) of entire double protofilament of each simulation, colored and numbered according to filament and replicate number. Dashed line represents the mean filament bSASA from microsecond-scale ATP-bound filaments (Fig. S2A). bSASA was lower in G66C simulations and the G45C simulation with lower twist angle (light blue).

**
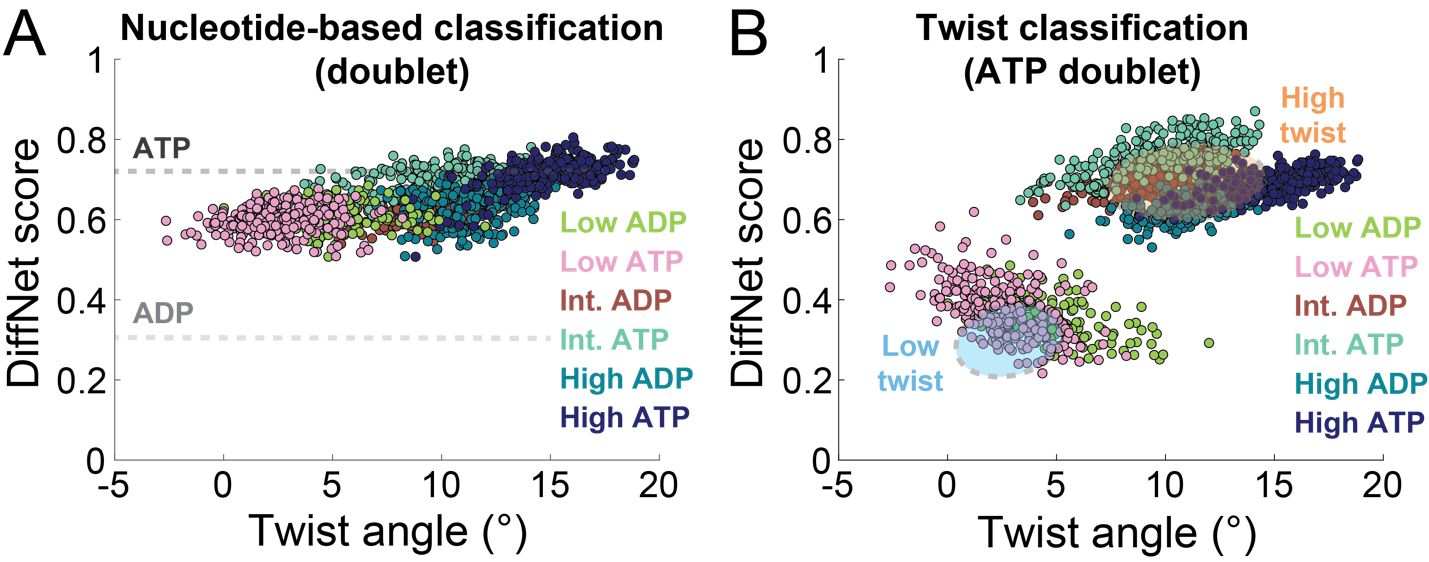
**

**Figure S11: DiffNet predicts twist states of hydrolysis mutants, but not nucleotide identity.**

1. Nucleotide-based DiffNet from Fig. S4 did not predict the bound nucleotide from simulations mimicking hydrolysis through nucleotide replacement (Fig. 4). Shown are DiffNet scores for frames of simulations initialized in a twist state from the first simulation in Fig. 1C with a low, intermediate (Int.), or high twist angle bound to either ADP or ATP. Also shown are the mean scores from DiffNet training (Fig. S4B) for the ADP-bound (light gray dashed line, mean = 0.3) and ATP-bound (dark gray dashed line, mean = 0.7) double protofilaments.
2. Twist-based DiffNet from Fig. 3 predicted twist states of the simulations in (A). Colored ellipses represent the distributions of DiffNet scores of the training data for high (orange) and low (blue) twist states (Fig. 3A,B).
